# Using Virtual Learning to Build Pediatric Palliative Care Capacity in South Asia: Experiences of Implementing a Teleteaching and Mentorship Program (Project ECHO)

**DOI:** 10.1200/GO.20.00481

**Published:** 2021-02-08

**Authors:** Megan Doherty, Spandana Rayala, Emily Evans, Jennifer Rowe, Vineela Rapelli, Gayatri Palat

**Affiliations:** ^1^ Children's Hospital of Eastern Ontario, Ottawa, Ontario, Canada; ^2^ Pain Relief and Palliative Care Society, Hyderabad, Telangana, India; ^3^ Faculty of Medicine, University of Ottawa, Ottawa, Ontario, Canada; ^4^ MNJ Institute of Oncology, Hyderabad, Telangana, India

## Abstract

Palliative care is an important component of pediatric cancer treatment that provides holistic support for children and their families. In low- and middle-income countries, where 98% of the children needing palliative care reside, access to palliative care services is often very limited. Training opportunities for healthcare professionals are essential to improve access to palliative care in these settings. Virtual learning, which brings training and mentorship directly to learners, can improve access to educational opportunities for staff in resource-limited settings. In this report, we describe a novel and evolving model of building pediatric palliative care (PPC) capacity in South Asia. We describe the design, implementation, challenges, and subsequent modifications of our program, as well as the impact of the program for participants and for PPC service delivery in South Asia. Our teleteaching and mentoring program (Project ECHO) [Extension for Community Healthcare Outcomes] consisted of biweekly videoconference sessions with didactic teaching and case-based discussions. The program focused on engaging participants in meaningful learning by focusing on opportunities for participant interaction through teachings and case discussions. Participants identified the program as particularly beneficial for improving their knowledge and confidence in managing seriously ill children. Project ECHO is a novel model of building PPC capacity that is suitable for resource-limited settings. Key modifications to the Project ECHO model include a course-specific leadership team, developing learning plans to address the specific learning needs of participants, focusing on ensuring learner participation during sessions, and using social media and electronic resources to create opportunities for further learning outside of ECHO sessions. These adaptations may improve the efficacy of Project ECHO and others using virtual learning programs in resource-limited settings.

## INTRODUCTION

There is a significant global need for children's palliative care, with an estimated 21 million children needing palliative care annually.^1^ Developing palliative care programs in resource-limited settings is a global priority since 98% of the children requiring palliative care reside in low- and middle-income countries (LMICs).^2,3^

**CONTEXT**

**Key Objective**
How can we develop an effective virtual pediatric palliative care (PPC) capacity building program suitable for resource-limited settings using the Project ECHO model?
**Knowledge Generated**
Project ECHO can be modified to include a leadership team with an in-depth understanding of the local healthcare situation and learning needs of participants, training for course facilitators on specific techniques that increase learner participation, and using familiar social media channels and electronic resources to support participants to engage in further learning outside of ECHO sessions. This capacity building program is highly valued by participants and supports the development of new PPC programs in the region.
**Relevance**
Using an adapted Project ECHO education model is a valuable capacity building strategy that supports healthcare providers to develop new knowledge and skills and mentors them to develop new PPC programs in resource-limited settings.

A lack of education about palliative care among healthcare providers is a significant barrier to improving palliative care availability.^4,5^ Healthcare providers may lack knowledge of how to assess and treat pain and other symptoms, and a recent survey of physicians providing cancer care in Bangladesh found that the majority of physicians were unaware of the potential therapeutic benefits of morphine for pain management and did not feel adequately trained to prescribe morphine for this indication.^6^

Online education has been suggested as an effective strategy for disseminating specialized training in LMICs, and a recent review of teleteaching for health professionals concluded that educational outcomes were as good as traditional in-person teaching methods.^7^ Using virtual training can also address the challenges of staff needing to take time off and to travel to attend education programs.^4,8,9^

**FIG 1:**
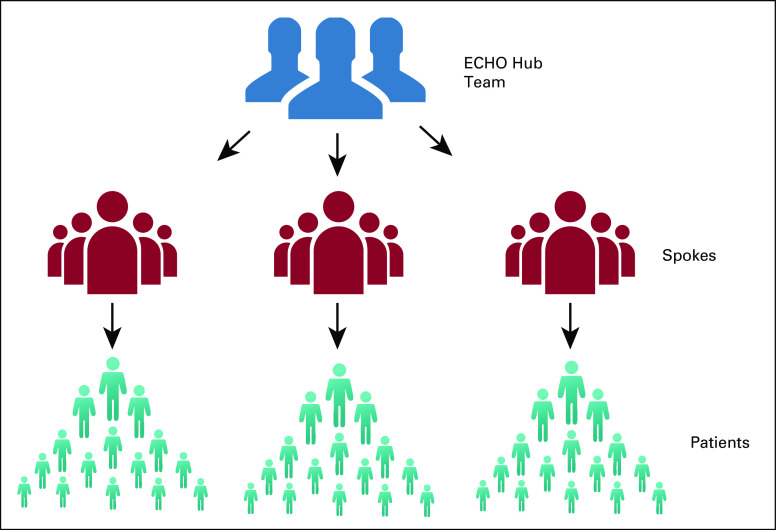
Hub and spoke model of Project ECHO (Extension for Community Healthcare Outcomes).

Project ECHO [Extension for Community Healthcare Outcomes] is an online technology-enabled capacity building model that focuses on improving community-level healthcare providers' knowledge and skills through teaching and mentorship.^10^ In Project ECHO, multipoint videoconferencing is used to connect local healthcare providers with specialists at a hub site (Fig 1). ECHO sessions are conducted at regular intervals (eg, weekly or biweekly) and follow a structured format of didactic teaching and case presentation and discussion.

Despite the proposed benefits of online learning in medical education, there can be significant challenges when implementing e-learning. A recent review of the topic, focused on resource-limited settings, identified the lack of face-to-face interaction as a significant challenge to engaging in interactive discussion, which is critical for learning.^11^ Additionally, the authors noted that virtual learning programs should be modified to be culturally relevant and to fit with healthcare realities of a particular country.^11^

## SETTING AND POPULATION

A recent study estimating the global need for pediatric palliative care (PPC) projected that 4.25 million children in India need palliative care at any one time, including 1.63 million who require specialized palliative care.^1^ Almost 80% of the children with cancer in high-income countries are cured; however, in LMICs, difficulties accessing diagnostic facilities and curative cancer treatment lead to survival rates that may be as much as four-fold lower.^12,13^

### Palliative Care in India

India has a universal publicly funded healthcare system that is provided by the central and state governments. Government hospitals provide essential treatments for free or at minimal charge; however, the government system is underfinanced and lacks adequate staff and resources to address the needs of the vast number of patients seeking medical care.^14^ The availability of palliative care also varies widely across India, with 19 of 36 states having no known palliative care activity.^14^ The state of Kerala has particularly well-developed palliative care services, which account for 90% of palliative care programs within the country despite having only 3% of the Indian population.^15,16^ Outside of Kerala, the majority of palliative care services are supported by nongovernmental organizations and are located within urban areas, leading to significant gaps for the majority of the population who reside in rural areas.^16,17^ Even in urban areas, most palliative care services are focused on adult patients, with very few dedicated pediatric programs; outside of Kerala, we are aware of only four pediatric-focused programs: Hyderabad, Mumbai, Delhi, and Pune.^14^

### Palliative Care Education in India

In recent years, several formal palliative care training programs for physicians and nurses have been developed, but none are focused on pediatrics.^16^ Palliative care training has also not been systematically incorporated into undergraduate training programs for physicians or other health professionals.

A recent study of pediatric postgraduate trainee physicians in South India found that 77% were uncomfortable discussing palliative care with families, yet most of those surveyed (87%) were very interested to improve their knowledge and skills in this area.^18^ Providing specialized training for primary care practitioners, nurses, and other professionals in rural settings has been identified as a priority to improve access to palliative care in India.^15,16^ Given its vast geographical area and largely rural population, technology-enabled learning provides a simple and economical solution for improving access to PPC education in India. Results from a recent survey study of Indian palliative care clinicians also identify high levels of interest in online training, suggesting that this form of intervention would be well-received.^19^

In this report, we describe the design, implementation, and impact of a new and evolving model of capacity building in PPC through an education program based on the Project ECHO model (ECHO PPC). We discuss the key steps in program development as well as our adaptations of the Project ECHO model to address the specific features healthcare professionals in India and in South Asia more broadly. Our experiences can guide other groups seeking to develop palliative care programs for health professionals in resource-limited settings.

## DEVELOPING AND IMPLEMENTING ECHO PPC

We selected the Project ECHO model that is specifically designed to reach healthcare workers in remote and underserviced areas, locations where many children needing palliative care in India reside. Our program was developed jointly by a local palliative care society (Pain Relief and Palliative Care Society, Hyderabad, India) and a nongovernmental organization (Two Worlds Cancer Collaboration, Vancouver, Canada), through the existing partnership providing palliative care education and clinical support in Hyderabad, India.

The initial focus of ECHO PPC was to build the capacity of healthcare professionals in India to provide PPC, and over time, our geographical scope has expanded to all of South Asia. Since PPC requires a multidisciplinary team approach, we invite physicians, nurses, psychologists, pharmacists, physiotherapists, social workers, and counsellors to participate in ECHO PPC programs.

Over the past 2.5 years, we have conducted a series of ECHO PPC courses, tailored to the needs of various groups of target learners. Table 1 lists the completed and upcoming courses. Topics for selected courses are shown in Appendix Table A1. We tracked the development of new PPC services in South Asia through our network of participants and professional networks.

**TABLE 1:**
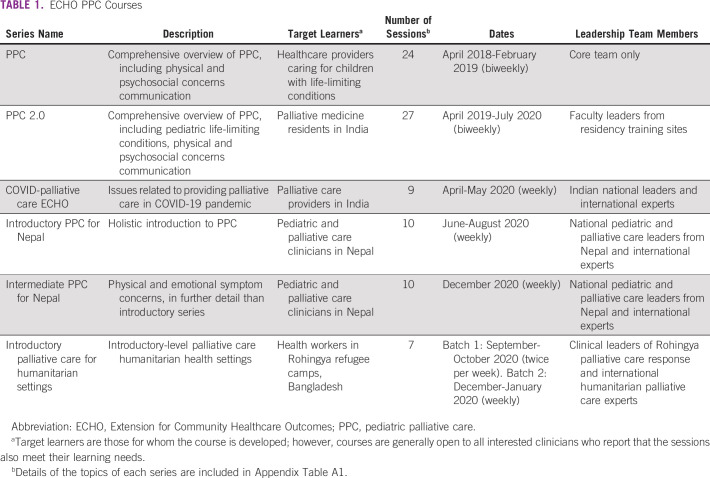
ECHO PPC Courses

For assessment of the program's impact, we invited learners from the first ECHO PPC program by e-mail to participate in an online survey to explore their experiences with the program. Demographic and professional practice characteristics of study participants were collected. This study was approved by the Children's Hospital of Eastern Ontario's ethics board, approval number 17/201X. Written informed consent was obtained from all study participants.

### Key Steps and Modifications

During development and evolution of these courses, we developed a number of innovations and adaptations to Project ECHO, which are highlighted in Table 2.

**TABLE 2:**
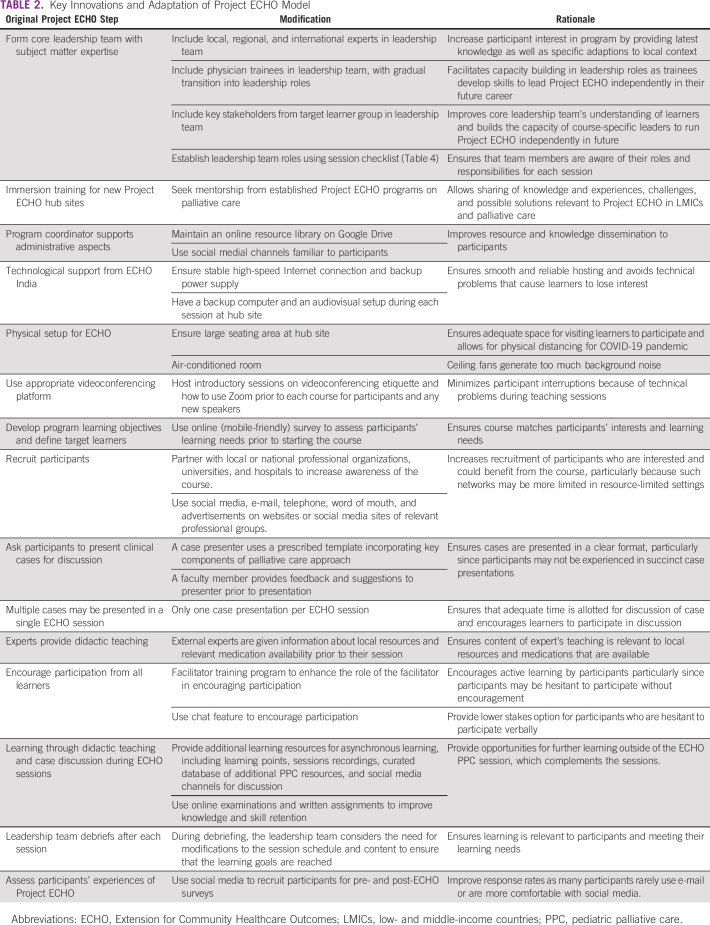
Key Innovations and Adaptation of Project ECHO Model

### Preparation Phase

#### Leadership and administrative support teams.

The core leadership team includes experienced palliative care physicians and other experts from India and Canada. We also include physician trainees, initially as observers on the leadership team, with a gradual transition into leadership roles as their skills increase. For each individual course, course-specific leaders are added to the core leadership team. These members are generally leaders or key stakeholders representing the target learners for a particular course.

We complimented the original mandatory training for new Project ECHO sites from Project ECHO (India) with additional mentoring from established Project ECHO leaders specific to palliative care from the Trivandrum Institute of Palliative Sciences–Pallium India and Hospice UK.

The program coordinator facilitates administration of the program by sending reminder and summary messages (via e-mail and WhatsApp) to participants and speakers, maintaining an online resource library (on Google Drive), and uploading session recordings to a video-sharing website for participants to access.

We developed a checklist (Table 3) of the leadership and administrative roles for each ECHO PPC session. This checklist is reviewed during the weekly preparation meeting, helping to ensure that leadership, administrative, and technical staff are aware of the specific roles and responsibilities for a particular week, which is particularly relevant given that the leadership team is often spread across several continents.

**TABLE 3:**
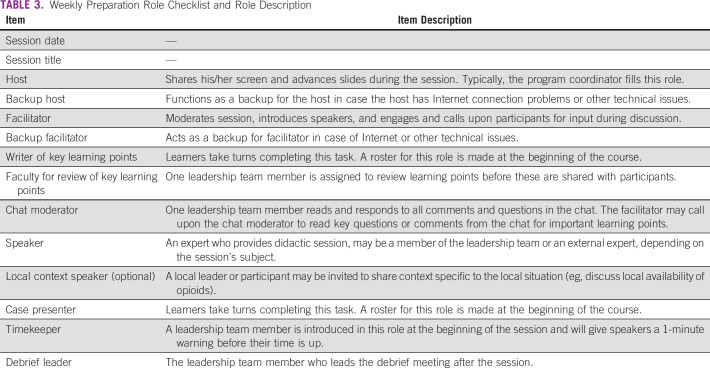
Weekly Preparation Role Checklist and Role Description

#### Technical considerations.

ECHO India provided on-site technical support with program launch and continues to provide support remotely. An information technology technician is employed to facilitate technical management of each session at the hub site. A stable high-speed Internet connection, backup power supply, and appropriate audiovisual equipment are needed to ensure smooth and reliable hosting from the main site, whereas participants can easily join from anywhere with a computer or mobile device. We use Zoom as the videoconferencing platform for our sessions, and our information technology technician hosts videoconferencing practice sessions for participants and speakers to minimize technical problems during sessions.

#### Determining program goals and curriculum design.

The leadership team identifies target learners and their specific learning needs through discussions and an online survey to assess learning needs prior to starting the course (an example of this survey is included in the Data Supplement). Topics that are frequently of high interest to participants are shown in Table 4.

**TABLE 4:**
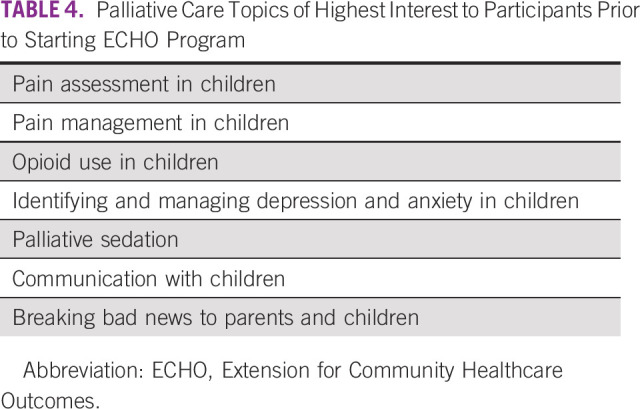
Palliative Care Topics of Highest Interest to Participants Prior to Starting ECHO Program

#### Participant identification and recruitment.

Recruitment strategies are adapted to the specific course and may include social media, e-mail, telephone, word of mouth, and advertisements on websites and social media sites of relevant local or national professional organizations, academic institutions, and healthcare facilities, such as Pallium India, the Indian Association of Palliative Care, the International Children's Palliative Care Network, and eHospice.

### Implementation

#### Session format.

The main components of each session are described in Table 5. A case presentation template is used to provide a simple and standardized format to facilitate clear case presentations from participants (Appendix Table A2). During each session, a clinical expert provides a short didactic lecture. The expert may be a member of the leadership team or an external expert depending on the subject.

**TABLE 5:**
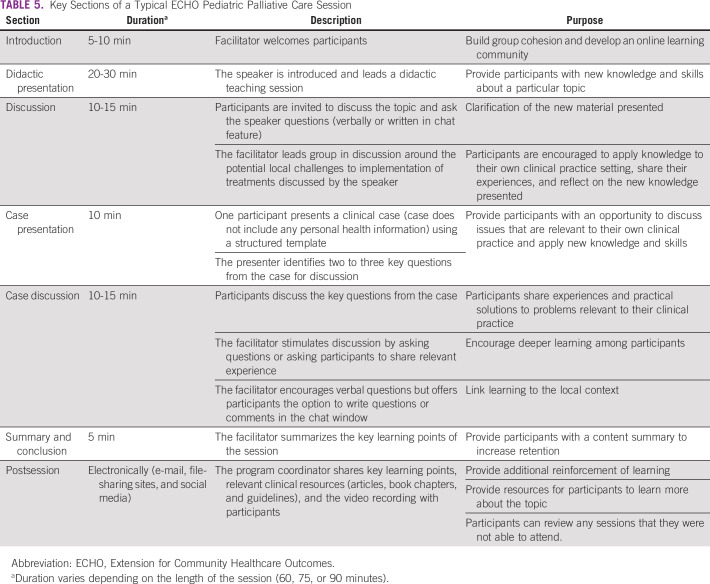
Key Sections of a Typical ECHO Pediatric Palliative Care Session

#### Facilitator.

The facilitator (a member of the leadership team) welcomes participants and introduces each component of the session, ensuring that the session flows smoothly, minimizing pauses between sections of the session. The facilitator pays particular attention to encouraging participation, using questions directed at specific participants and comments that guide the flow of the discussion toward key teaching points. Two large screens at the hub site to visualize of all participants simultaneously, which allows the session facilitator to observe participants' expressions and encourage greater learner engagement. We have developed a facilitator training program to provide structured guidance for new facilitators.

#### Chat feature.

Participants are also encouraged to use the chat feature in Zoom as an additional way of interacting. Comments and questions in the chat may be read aloud by the facilitator. A member of the leadership team is assigned to moderate the chat each week and respond to questions that may not be answered verbally during the session. We find that the chat discussion often complements the audio discussion and that there is often no time to discuss all the issues raised by participants. There may also be questions in the chat that are not relevant to the larger group of learners or not relevant to the session's topic, which can instead be answered individually by the chat moderator.

#### Learning materials.

Participants can access a variety of learning materials outside of ECHO PPC sessions, which are shared via e-mail and social media. Social media channels are developed to allow participants to ask questions and interact with the new material outside of the sessions.

### Program Evaluation

#### Debriefing.

The leadership team conducts a short debrief immediately following each session to discuss progress toward the desired learning outcomes, and the team may make modifications to the session schedule and content on the basis of their discussion, continuously allowing improvements of the course to ensure it meets the learning needs of participants.

#### Survey of participants.

At the end of each course, we conduct an online survey of participants about program satisfaction; the barriers and enablers of participation; and their knowledge, skills, and self-efficacy in palliative care. Although a full discussion of the results of this evaluation is beyond the scope of this article, Table 6 shows a summary of responses from participants from the first ECHO PPC course. These participants describe the main benefits of participation as learning more about PPC, learning from the experience of other participants, and hearing about palliative care practices in different countries. Additionally, participants noted the benefit of an opportunity to learn and discuss relevant topics and how this increased their own motivation to continuing learning about PPC and their awareness of their own learning needs. We found that survey response rates were low when sent by e-mail; after the first course, we have modified our recruitment strategy to use social media.

**TABLE 6:**
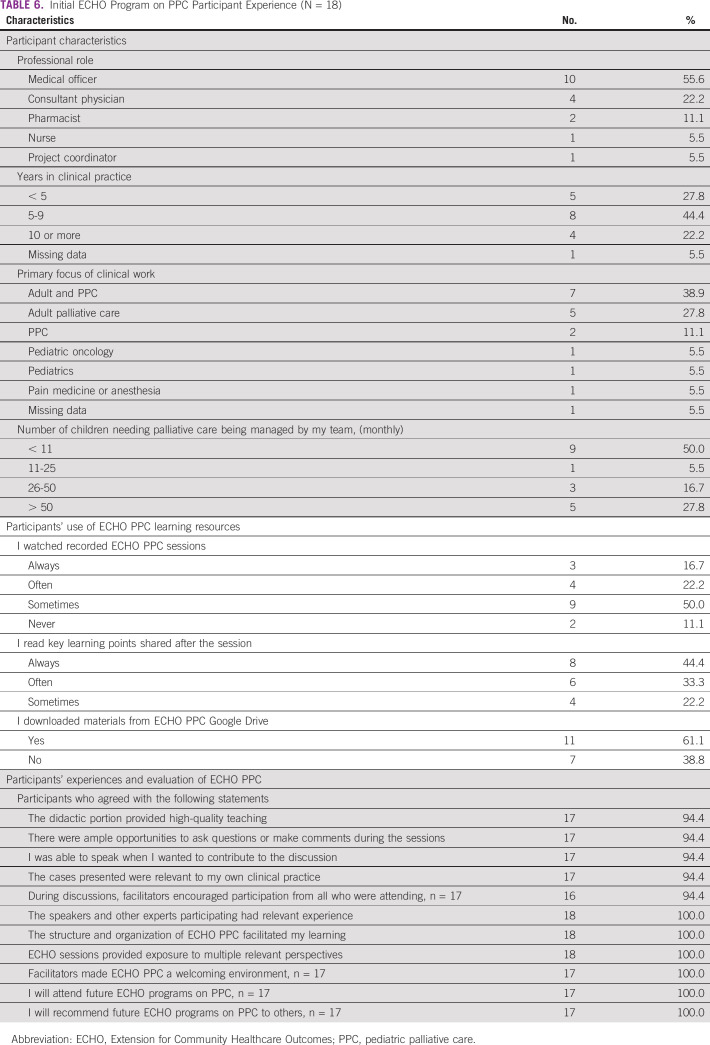
Initial ECHO Program on PPC Participant Experience (N = 18)

The main factors that facilitated participation include convenient session timing and duration, ease of accessing the sessions online, and reminder e-mails. The most significant obstacles of participation included Internet connection problems and busy work schedules. Participants were very motivated to attend further training, and all stated that they would recommend ECHO PPC to others.

#### Service delivery impact.

We are aware of the development of fifteen new services, including ten community-based palliative care services that are able to support children in India^8^ and Bangladesh^2^ and five hospital-based PPC services (one in India and four in Bangladesh), since 2018. Although the implementation of these services is not fully attributable to ECHO PPC, it is notable to consider that ECHO PPC may have supported the development of these services, particularly since healthcare providers from 11 of these programs have participated in ECHO PPC.

## DISCUSSION

We have described our experiences developing, adapting, implementing, and evaluating a novel capacity building program for PPC. ECHO PPC consists of regular (weekly or biweekly) virtual training sessions, incorporating didactic teaching from international experts and clinical case discussions. Our model of capacity building incorporates key principles from health professionals' education into its design and implementation, including focusing on participants' self-identified gaps in knowledge, providing opportunities for problem-oriented learning, and creating a supportive learning community.^20,21^

Our flexible and adaptive leadership structure includes a core leadership team and course-specific leaders and stakeholders. Previous e-learning courses have identified the importance of ensuring that all partners understand the local healthcare and educational systems of trainees.^22^ This structure allows our core leadership team to gain an in-depth understanding of the local medical culture and healthcare situation from the course-specific leaders.

Having a defined mission statement has been identified as an important step for health professional training programs.^23^ In our setting, we found that ensuring a unified course vision required a series of discussions between core and course-specific leadership team members to develop a common shared understanding and vision for the course. The program goals then guide the development of a learning plan to meet the educational needs of the particular learners, with weekly leadership team debriefing meetings to address newly identified learning gaps, which often become more apparent as the course progresses.

Understanding the practice characteristics and baseline knowledge of program participants has been identified as important to ensuring that training is at an appropriate level and allows training to build on participants' prior knowledge and skills.^23^ We developed a brief survey for new participants to learn about their professional and clinical practice characteristics. The survey data as well as participant feedback from previous ECHO PPC courses and the experiences of the leadership team inform the selection of didactic teaching topics for each course.

Incorporating case-based learning is an important aspect of the Project ECHO model since active learning and peer discussion are associated with improved learning outcomes.^24^ However, in a virtual environment, interactivity requires additional efforts since nonverbal speaking cues are often not visible to participants.^11,20,24^ We describe how the facilitator plays a key role in creating a supportive and encouraging learning environment in the virtual context. Facilitators use specific strategies to ensure that learners feel safe and comfortable and to minimize traditional healthcare hierarchies.^20^ These strategies include welcoming participants by name, understanding participants' clinical roles, and calling on specific participants to share their expertise and experiences. Facilitators may also invite participants to read aloud their comments or questions from the chat. Our preliminary survey findings suggest that this format provides a safe and welcoming environment for participants and encourages them to feel comfortable speaking.

An additional aspect of a supportive learning community is the availability of learning resources in a time and form that is convenient for participation. We found that participants frequently used ECHO PPC resources after the live sessions, with the majority watching session recordings (89%), reading key learning points (100%), and downloading database resources (61%). These findings suggest that learning from Project ECHO can be enhanced by expanding the learning environment beyond the individual Zoom sessions, which has not been discussed in previous descriptions or reviews of other Project ECHO programs.^25^ Since most learners in LMICs are using mobile devices (not computers) to access social media, it is important that electronic resources are suitably formatted for mobile devices.^26^

We used pre- and post-ECHO assessments of participants' knowledge, skills, and attitudes about palliative care to assess the impacts of ECHO PPC, which has been described in previous Project ECHO programs.^25^ There are limited data regarding the evaluation of Project ECHO outside of high-income countries, and we found that low survey response rates posed a particular challenge that may be particularly relevant in resource-limited settings. We initially used e-mail for survey distribution but found that distributing invitations and reminders via social media platforms has improved response rates.

In conclusion, Project ECHO is a novel model of building PPC capacity that is suitable for resource-limited settings. Key modifications to the Project ECHO model include a course-specific leadership team and a curriculum that addresses the specific cultural and healthcare system realities of each group of learners. The learning experience was further enriched by the use of facilitators to enhance learner participation during sessions and social media and electronic resources to create opportunities for further learning outside of ECHO sessions. These adaptations may improve the efficacy of Project ECHO and others using virtual learning programs in resource-limited settings.

## Figures and Tables

**TABLE A1:**
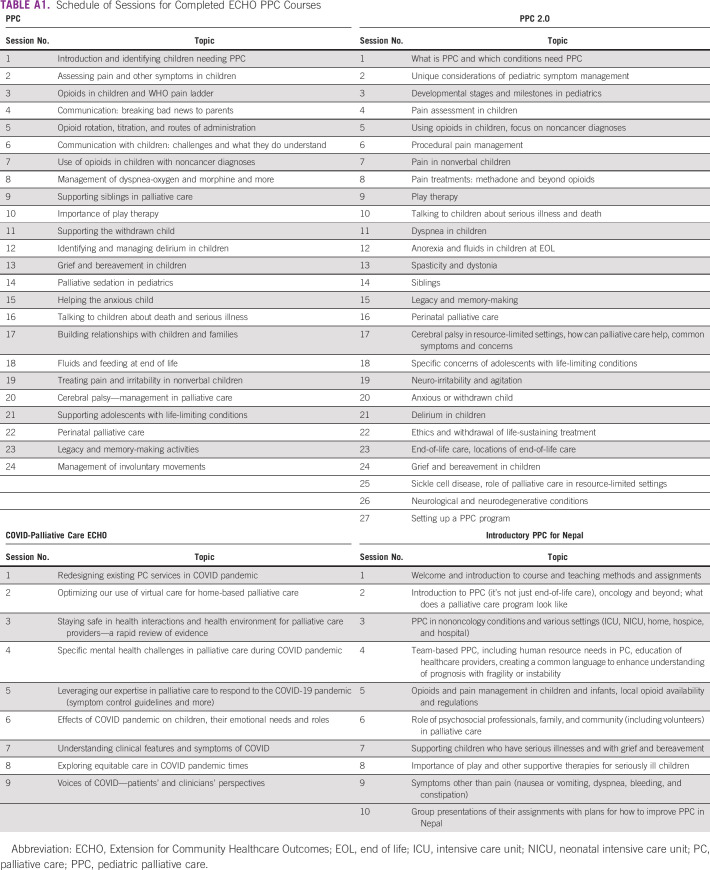
Schedule of Sessions for Completed ECHO PPC Courses

**TABLE A2:**
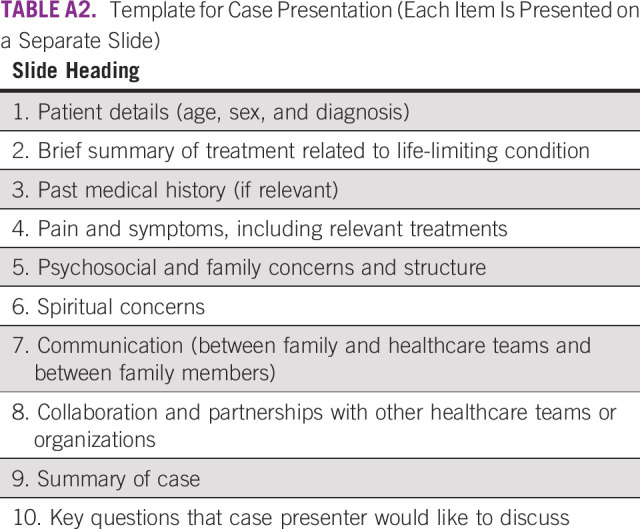
Template for Case Presentation (Each Item Is Presented on a Separate Slide)
